# Supplementary material for: Did Covid-19 make things worse? The pandemic as a push factor stimulating the emigration intentions of junior doctors from Poland: A mixed methods study
Source: PLoS One. 2024 Apr 16;19(4):e0301757. doi: 10.1371/journal.pone.0301757 (PMC11020858; doi:10.1371/journal.pone.0301757)
Supplement: Attachment — Submitted filename: Response to Reviewers.docx [file pone.0301757.s001.docx]

RE: PONE-D-23-08865

Did Covid-19 make things worse? The pandemic as a push factor stimulating the emigration of young doctors from Poland: a mixed methods study

PLOS ONE

Dear Editor,

Dear Reviewers,

Thank you very much for your work and comments, which we believe have helped us significantly improve the paper, particularly its clarity and structure. Here is a summary of the changes made, in the order in which they appear in the paper:

1. The abstract has been edited and quantitative results have been added (in line with the suggestion of Reviewer 1).
2. Our main terms are used consistently throughout the abstract and paper and are defined in the conceptual section. In line with migration literature, we speak of “migration intentions”, as well as “medical students” and “junior doctors”. We have decided that “junior” is a better translation than the previously used “young”, as a term that is more frequent and precisely defined in the academic literature in line with what we mean – persons who have already graduated from medical school but have not completed their specialisation and reached consultant or general practitioner level (in line with the suggestions of the Editor and Reviewer 2).
3. The whole first part (up to page 12) has been rewritten with the following aims in mind (in line with the suggestions of the Editor and both Reviewers):
a) to update the literature review and focus the narrative even more on the topic of factors pushing doctors to migrate, and on what is already known about the influence of Covid-19. Some other information regarding the situation of the health sector in Poland has been deleted, as not directly relevant;
b) to avoid repetitions.
4. Much detailed information has been added regarding our methods and respondents. We have also placed the data used in this study in a repository (OSF - https://osf.io/vkbnx/), so that the study may be replicated by other authors. (Editor and Reviewer 1) The section based on qualitative data has been rearranged to focus it more on our main topic.
5. The discussion and conclusions sections have been separated, and the conclusions synthesized (Reviewer 1). Limitations are dealt with at the end of the discussion (Reviewer 1). We have also made clearer what the theoretical contribution of our work is (Editor and Reviewer 2).
6. Finally, we have also used this opportunity to proofread the paper and make sure all references are in place.

Again, thank you for your time and helpful comments. We hope that the manuscript is now much more transparent, making our contribution clear.

Best regards,

Dominika Pszczółkowska

(on behalf of all the authors)
