# Supplementary material for: Did Covid-19 make things worse? The pandemic as a push factor stimulating the emigration intentions of junior doctors from Poland: A mixed methods study
Source: PLoS One. 2024 Apr 16;19(4):e0301757. doi: 10.1371/journal.pone.0301757 (PMC11020858; doi:10.1371/journal.pone.0301757)
Supplement: Attachment — Submitted filename: Response to Reviewers 2.docx [file pone.0301757.s002.docx]

Dear Editor, Dear Reviewers,

Thank you for again engaging with our article and helping to improve it. Given that the comments were specific to particular sections of the article, we respond to them point by point below. Line numbers in the answers below refer to numbering in the document with tracked changes.

*Reviewer #2: Thank you for this revised submission, it has much improved, yet, I feel it needs further work to make it of a publishable standard.

Here are my main concerns:
1. It is uncommon to have empirical contribution stated in the introduction, better to move it to conclusion.*

Thank you. The empirical findings have been removed from the introduction and are left only in the conclusion.

*2. Language needs tightening, it feels rushed e.g.:
100 'good ...' - what?*

In the sentence „The value a particular individual ascribes to a good”, „good” is not an adjective, as you seem to suggest. It is a noun, used in the meaning of „things for [sale](https://dictionary.cambridge.org/dictionary/english/sale), or the things that you own” (Cambridge Dictionary) or „something that has economic utility or satisfies an economic want” (Merriam-Webster Dictionary) e.g. access to schools may be a valuable good to one migrant but of no value to another. The sentence has been rephrased and examples have been added to make this clear.

*102 'destination' country?*

If I understand correctly, you are suggesting we should speak of destination countries and not only of destinations? The theory we write about here is not focussed on countries only. Lee (1966: 49) writes: “No matter how short or how long, how easy or how difficult, every act of migration involves an origin, a destination, and an intervening set of obstacles”. Choices made by doctors are not based only on factors related to particular countries (e.g. legal framework) but also factors related to destinations (e.g. safety in the streets of a city or access to cultural events). We do not see the reason for limiting the discussion here to destination country choice.

*284 'doctors' - meaning the participants?*

Changed to medical students, thank you (line 319)

*451 which previous scholarship?*

Cited in the next sentence (line 588).

*462 how?*

Clarified in the text (lines 596-7), where the sentence now reads “It introduces a new element into the discussion on the determinants of high-skilled migration, extending the list of possible push factors that need to be considered.”

*decide whether to use LGBT or LGBTQ or LGBTQ+*

We now only use LGBTQ (line 473).

*3. The paper seems difficult to follow at times, e.g.:
330-331; 343 - yes, and what did they say?*

Edited to make these lines clearer.

*4. The qualitative part i.e. the interview extracts seem to be just described in the analysis section - state what is particularly interesting and relevant to healthcare staff as it appears that most of what is reported could apply to the general public too.*

This section has been significantly rearranged and edited to better demonstrate what the qualitative interviews have contributed to our knowledge regarding junior doctors (lines 382-481).

*5. The discussion section doesn't offer a discussion - a synthesis of the results is needed here.*

Added at the beginning of the discussion section (lines 583-588)

*6. The authors white of 'socio-political factors in the context of junior doctors' emigration intentions' - but most of the participants don't intend to emigrate, right? If so, then how is this the 'context'? Perhaps this should be rephrased as at the moment it seems that the paper is about covid's 'impact on migration decisions' but did they even consider emigration before they were asked about it?*

The sentence quoted above has been rephrased, as indeed the word ‘context’ was not the best way to put it. Here, we would like to offer some background data and knowledge from the field of migration studies to respond to the issues raised above.

Regarding the socio-political factors: in response to the survey question “To what degree do the following factors push you to leave Poland?” a huge majority of respondents found socio-political factors very encouraging (116 respondents) or encouraging (34) to leave. This was out of a total of 181 persons who were asked this question (all those whose likelihood of migration was not 0 on the 11-point Likert scale). Furthermore, as demonstrated in Table 2, the strongly positive and positive responses were correlated with higher migration intentions.

Regarding whether most of the participants intend to emigrate: given that we asked them to judge the probability of migration on the Likert scale (0-10), it would be simplistic to speak about what most intend to do, especially for people who were around the middle of the scale. However, it is very unlikely that they never thought about it before we asked – Poland has long been an emigration country, and especially the history of medical migrations quoted in the text means that it is very unlikely that a medical student would not know others who have migrated.

*Reviewer #3: A key strength of this study is the use of mixed methods with the same participant group, providing both quantitative data on migration intentions and qualitative insights into reasons behind them. However, the survey methodology requires clarification. More details are needed on survey response rate and representativeness. How many participants provided contact info for interviews and what was the response rate? Without this information, it is difficult to assess potential nonresponse or selection bias.*

This information has been added (lines 244-252, 256-266)

*The authors should also clarify inconsistent labeling of the interview participants as both "medical students" and "junior doctors." It is applicable to the results section: the authors, when analyzing the quantitative survey results (lines 317-319), used the concept of "junior doctors" while the participants were still medical students at the time of the research.*

Thank you for pointing out this mistake. Although they would most likely be migrating as junior doctors after graduation, at the time of the survey, all participants were indeed medical students. They were junior doctors at the time of the interviews, which took place several months later. This has been clarified in several points in the text (e.g. lines 306, 318, 321).

*Regarding generalizability, the lack of information on survey respondent distribution across different universities is a limitation.*

This information has been added (lines 248-252).

*As social media recruitment was used, respondents beyond the targeted university may have participated without the authors' knowledge, hampering generalizability.*

The fact that the survey was taking place was announced on social media. However, the link to the survey itself was not publicized this way. It was only distributed via e-mails sent to 6^th-^year students. It was thus not possible to participate only by seeing the advertisement on social media. We made this clearer in the text as it was indeed phrased misleadingly (lines 236-7).

*Further details on number of respondents by university year would also be beneficial.*

This is a misunderstanding. All students participating in the survey were in their 6^th^ year, so there is no division by university year. To clarify this, we have reiterated in the text that all survey respondents were in the final (6^th^) year of their studies. We have also added the number of students in each school and the number of respondents (lines 244-252).

*The small interview sample raises similar concerns, as reasons for selecting 6 men and 3 women are unclear given gender distribution differences in the medical student/doctor population.*

Indeed, the gender distribution of the interview participants is not representative of the whole population of 6^th-^year students and does not correspond to the gender distribution of the participants of the survey. As we have now explained in the text (lines 260-272), the interview participants were drawn randomly from among those who agreed to continue their participation in the research and provided contact details. The procedure of drawing the respondents was focused on recruiting participants from all schools, as these ranged from Warsaw to the significantly smaller city of Białystok. Since only 9 of the 32 participants who received e-mail requests chose to participate, we did not have full control of the gender balance. However, as is the case in qualitative research, the aim here was not to have a representative sample of respondents but rather to gain a deeper understanding of the answers provided in the survey. In the survey, we did not observe significant differences in answers depending on gender.

*While the study provides useful exploratory data, the limitations hinder broad applicability of the conclusions. I suggest revising the framing to focus more precisely on this cohort rather than generalizing. Additional details on sampling and methodology are needed to assess rigor and potential biases. Discussion of study limitations should also be expanded. Overall, this is an interesting research question but requires refinement to match the appropriate scope of inference.*

All of the above details were added, and we make it clearer at many points in the text that the focus is on medical students (and junior doctors, in the case of interviews). More discussion of the limitations was added (lines 244-252, 256-266, 609-617).

*More detail suggestions:
Defining key terms like “migration intention,” “medical students,” and “junior doctors” should be moved to the introduction or methods section.*

Moved to the introduction, thank you (lines 91-95).

*Line 169 – bracket needed*

Added.

We are grateful for all the above comments, which have helped to avoid any misunderstandings, making the text clearer regarding its findings and contribution. Additionally, the text has been proofread by a native speaker to improve its readability and the quality of English.

Best regards,

The Authors
