# Supplementary material for: Did Covid-19 make things worse? The pandemic as a push factor stimulating the emigration intentions of junior doctors from Poland: A mixed methods study
Source: PLoS One. 2024 Apr 16;19(4):e0301757. doi: 10.1371/journal.pone.0301757 (PMC11020858; doi:10.1371/journal.pone.0301757)
Supplement: Attachment — Submitted filename: Response to Reviewer with version 4.docx [file pone.0301757.s003.docx]

Dear Editor,

Dear Reviewers,

Thank you for again engaging with our article, and for your encouraging comments and suggestions for further improvements. They have all been reflected in the text, along with some minor language changes elsewhere. One new position has also been added to the bibliography (hence the change in the numbering of references). We discuss the changes below after each of your comments.

Best regards,

The authors

Reviewer #2: Thank you for this revised submission. It has much improved, although, I still have a few issues with it:

1. 'Migration decisions have a very well established basis' - this is a very problematic statement in my view, which is due to existing scholarship which says otherwise and also due to how definitive it is. That said, I'm not here to argue my point of view and it may just be that, a point of view.

We added “in the case of medical students” to clarify. This is what we meant – that the migration intentions of this group, not people in general, have a well-established basis, more than other more spontaneous ‘intentionally unpredictable’ or ‘liquid’ migrations which were found to be characteristic of early post-EU accession migration from Poland.

2. line 83 - 'pandemics' reads awkward; also, did I miss this lit review of the influence of health crisis the authors refer to? Additionally, this contradicts line 109 where it says that the review only includes publications from the ages 2009-2019 which is pre-covid.

This has been changed to better reflect the order of further sections of the paper and make clear that we are reviewing research on the effects of the Covid-19 pandemic (not ‘pandemics’ in general). Line 109 /110 has been changed to reflect the fact that the review indeed covers all the recent literature up to 2023.

3. line 178 - 'influx' is a negative word with pejorative connotations and thus I wouldn't use it when describing those seeking asylum due to an armed conflict - there's a debate on the new acceptable terms within migration studies that the authors may wish to follow.

We have changed ‘influx’ to ‘arrival’ here and in the conclusions. We have unified our terminology to ‘war refugees’ throughout the text. Indeed, we have been following the discussion regarding the terms used to describe people fleeing Ukraine. We believe ‘war refugees’ is the most appropriate (and concise) term to describe these people, who have had to seek protection outside of their country but due to the implementation of the EU Temporary Protection Directive are usually not applying for refugee status.

4. I'd be very interested to learn the ages of the respondents but I recognize that this isn't necessary for this submission but it would give more context and enable better understanding of the results.

We did not ask this question, as it is known from medical school data that a huge majority of students are of about the same age (23-26 when in their 6^th^ year). To know their life stage, we asked if they had children, and only six people in the sample did. A majority were also not married, some were in relationships. This is just for your information, we did not add this to the article as it does not seem very relevant.

5. line 372 - 'resumed' ?

Changed to “summed up”.

6. line 441 - it led to spreading the word/publicizing details in national media about women dying due to a lack of access to legal abortion and not to publicized cases

Changed to “which led to the media reporting cases of pregnant women dying”. We want to say that these women really died and that their deaths were discussed in the media. Legal cases against doctors are ongoing, so it is still prudent to say that “perhaps” their lives could have been saved.

7. line 470 - I wouldn't call it 'under-recognized factor of hate speech' - see a list of available resources that came up through just a quick search:

<https://blogs.bcm.edu/2023/07/14/hate-speech-in-healthcare/>
<https://czasopisma.uwm.edu.pl/index.php/mkks/article/view/6410>
<https://www.mirecc.va.gov/visn16/working-with-patients-who-use-hate-speech.asp>
<https://www.taylorfrancis.com/chapters/edit/10.4324/9780429201813-6/bad-bedside-manner-sheri-wells-jensen-claire-wells-jensen>

"Under-recognised” has been crossed out.

Reviewer #3: As I have reviewed the article in the first round, and had paid attention to necessary changes, which were incorporated by the authors in the subsequent submission, there are not too many comments on the reviewed text.

However, there are some suggestions:

It would be advisable to emphasize more clearly the justification for the necessity of conducting research in Poland – it is worthwhile to refer to the OECD report, providing specific indicators of the number of doctors/nursing staff per 1000 inhabitants in Poland.

The number of doctors has been added.

There is a question about conducting a literature review for the years 2009-2019? (10-years period?).

As has been mentioned above, this was a mistake on our part, as we also cite works from later years, and two from 2007-2008. This has been changed to “the recent literature (up to 2023)”.

There is a lack of a clear message/key presentation in the introduction of the literature review. Perhaps it is worth considering a division based on push and pull factors, factors related to different disciplines.

It would be difficult to fit some of the data cited, particularly surveys which asked about plans but not the reasons behind them, into the push-pull framework. However, this section has been edited slightly for clarity.
